# Supplementary figures and images for: The potential mechanism of Huangqin for treatment of systemic lupus erythematosus based on network pharmacology, molecular docking and molecular dynamics simulation
Source: PeerJ. 2025 Jun 26;13:e19536. doi: 10.7717/peerj.19536 (PMC12206403; doi:10.7717/peerj.19536)

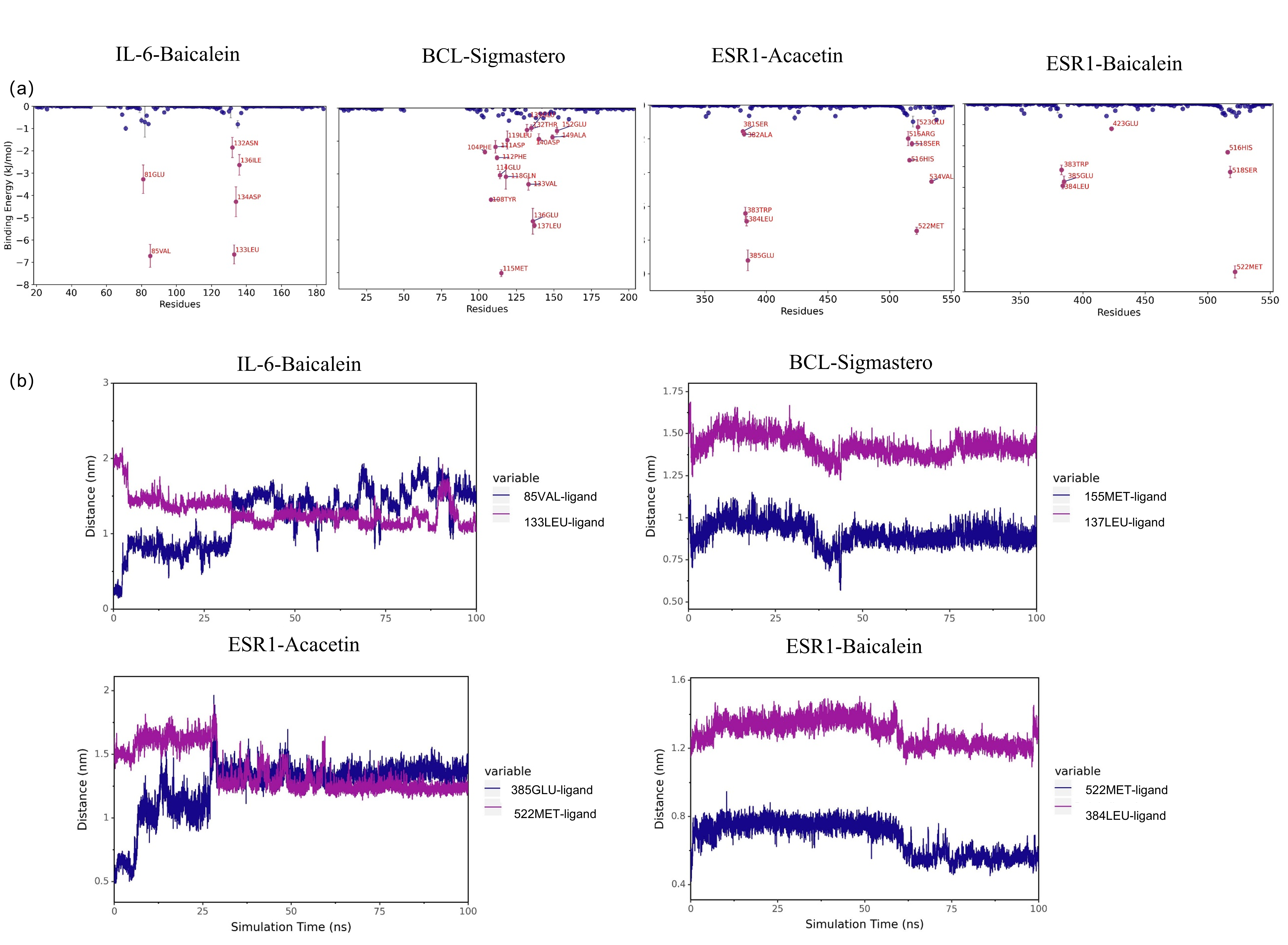

Supplement: Supplemental Information 6 — (a) the residues of the four complexes (b) the evolution of the distances between ligand features and the top2 residues. [file peerj-13-19536-s006.png]
